# Screening auf psychische Komorbiditäten in der Dermatologie: Erfolgreiche Implementierung eines Screenings auf psychische Komorbiditäten im Bereich der stationären dermatologischen Versorgung

**DOI:** 10.1007/s00105-020-04723-y

**Published:** 2020-11-12

**Authors:** Felix-Wilhelm Köster, Sebastian Kohlmann, Siobhan Loeper, Bernd Löwe, Stefan W. Schneider

**Affiliations:** 1 Klinik und Poliklinik für Dermatologie und Venerologie, Universitätsklinikum Hamburg Eppendorf, Martinistr. 52, 20246 Hamburg, Deutschland; 2 Klinik und Poliklinik für Psychosomatische Medizin und Psychotherapie, Universitätsklinikum Hamburg Eppendorf, Hamburg, Deutschland

**Keywords:** Patient Health Questionnaire‑4, Psychodermatologie, Ganzheitliche Medizin, Ängstlichkeit, Depressivität, Patient Health Questionnaire‑4, Psychodermatology, Holistic medicine, Anxiety, Depression

## Abstract

**Hintergrund:**

Ängstlichkeit und Depressivität sind bei Patienten im Bereich der stationären somatischen Versorgung weit verbreitet. Gerade in der Dermatologie, wo oft eine Behandlung von chronischen Erkrankungen erfolgt und die Gefahr der Stigmatisierung durch die Gesellschaft besonders groß ist, treten psychische Störungen bei fast jedem dritten Patienten auf. Dermatologische Erkrankungen und psychische Störungen stehen oft in negativer Wechselwirkung und führen zu gesteigerter Morbidität. Obwohl dermatologische Leitlinien eine Früherkennung empfehlen, wird dies in der Praxis oft unzureichend umgesetzt.

**Methodik:**

Wir demonstrieren die Etablierung eines einfachen Screenings im Bereich der stationären dermatologischen Versorgung auf psychische Komorbiditäten anhand eines kurzen Fragebogens, dem sog. Patient Health Questionnaire‑4 (PHQ-4), der 4 Fragen zur Ängstlichkeit und Depressivität stellt. Wird hierbei ein bestimmter Punktwert erreicht, erfolgt die automatische Anforderung eines psychosomatischen Konsils. Dadurch können eine Entlastung des Patienten sowie die notwendige ganzheitliche Behandlung erfolgen.

**Ergebnisse:**

Im Jahr 2019 wurden in unserer Klinik 83 % aller stationären Patienten mittels PHQ‑4 gescreent und 98 psychosomatische Konsile generiert.

**Diskussion:**

Unsere bisherigen Erfahrungen zeigen den Nutzen des Screenings bei geringem zeitlichem Mehraufwand, sodass wir eine flächendeckende Einführung in der stationären dermatologischen Versorgung empfehlen.

## Abstract (en)

**Background:**

Anxiety and depression are common in the field of inpatient somatic treatment. Especially in dermatology in which the treatment of chronic diseases is very common and the risk of stigmatization by society is particularly high, mental disorders appear in every third patient. Dermatological diseases and mental disorders often negatively interact with each other leading to increased morbidity. Dermatological guidelines recommend early detection, but this is often not adequately done in practice.

**Materials and methods:**

We present the implementation of an easy screening for mental comorbidities in the field of inpatient dermatological treatment by using a short questionnaire. This so-called Patient Health Questionnaire‑4 (PHQ-4) consists of four questions regarding anxiety disorders and depression. Upon reaching a certain number of points, a psychosomatic consult is automatically requested. As a result the patient’s stress is relieved and the necessary holistic treatment becomes possible.

**Results:**

In 2019, 83% of inpatients in our clinic were screened using the PHQ‑4; 98 psychosomatic consults were performed.

**Conclusion:**

Our findings so far have shown the benefit of the screening with a minimal investment of time. We recommend its comprehensive use in the field of inpatient dermatological treatment.

Psychische Komorbiditäten stellen ein bedeutendes Problem in der Behandlung von dermatologischen Patienten dar. Sie sind oft nicht nur Folge der dermatologischen Erkrankung, sondern verschlechtern diese zusätzlich. Eine Detektion einer psychischen Begleiterkrankung und Einleitung einer adäquaten Therapie wäre somit im Rahmen der dermatologischen Behandlung wünschenswert. Leider mangelt es bisher an einem praktischen Screening auf psychische Komorbiditäten in der Dermatologie.

## Hintergrund

Depressive Störungen und Ängstlichkeit treten aufgrund des oft chronischen Verlaufes gehäuft bei Patienten mit dermatologischen Erkrankungen wie etwa Psoriasis, atopischer Dermatitis, chronischem Juckreiz, Vitiligo oder auch Hidradenitis suppurativa auf [1, 2, 6]. Die psychische Belastung kann dabei sogar zu Suizidalität führen [7, 9]. Insgesamt wird die Komorbidität von psychischen Störungen bei dermatologischen Erkrankungen auf ca. 30 % geschätzt [4]. Eine psychische Belastung wirkt sich wiederum z. B. durch häufiges Kratzen oder schlechte Compliance negativ auf das Krankheitsverhalten aus und erschwert die dermatologische Behandlung [11]. Die Früherkennung von psychisch belasteten Patienten wird folglich durch zahlreiche Fachgesellschaften empfohlen [10]. Die Umsetzung dieser Empfehlung in der dermatologischen Routine erfolgt aber selten, da es an bedarfsgerechter, psychotherapeutischer Versorgung mangelt. Die unbehandelte psychische Belastung der Betroffenen stellt dabei oft eine Belastung für die dermatologischen Behandler dar.

Zur Früherkennung von psychischen Störungen existieren gut validierte Fragebögen, die frei verfügbar sind und aufgrund ihrer Kürze schnell einen zuverlässigen Screeningbefund liefern können. Der Patient Health Questionnaire‑4 (PHQ-4) kann mittels von nur 4 Fragen mit hoher Sensitivität (83 % für depressive Störungen, 76 % Panikstörung) und hoher Spezifität (depressive Störungen 90 %, Panikstörung 81 %) psychische Störungen detektieren [5, 8]. Die Erhebung des Fragebogens dauert nur ca. 1–2 min.

Vor dem Hintergrund der Versorgungslücke und dem Leid der Betroffenen stellt sich die Frage, wie eine effiziente Früherkennung und Behandlung von Patienten mit psychischen Komorbiditäten erfolgen kann.

## Methodik

**Figure 1:**
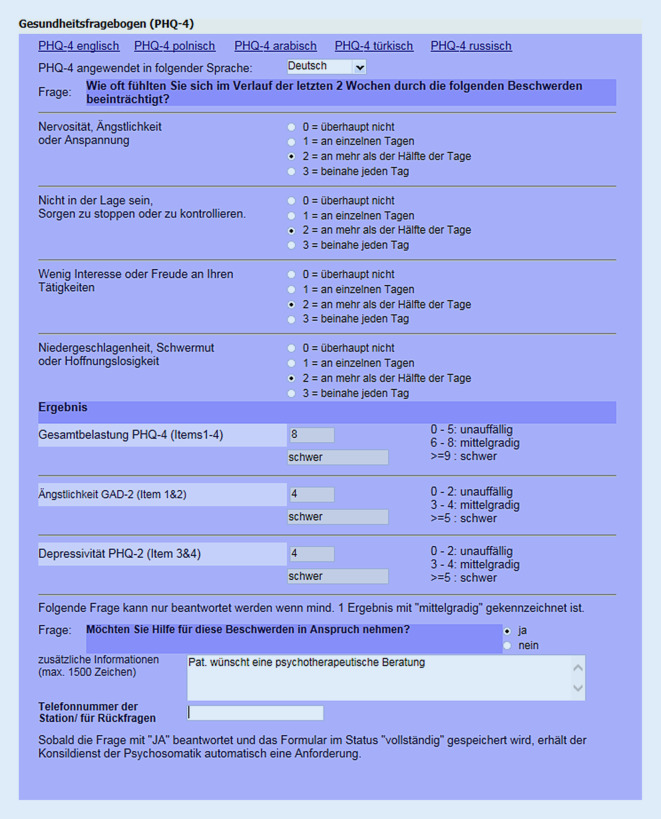

Seit Januar 2018 besteht für das medizinische Personal der Klinik für Dermatologie und Venerologie des Universitätsklinikums Hamburg Eppendorf die technische Möglichkeit der Erhebung des PHQ-4-Fragenbogens in der elektronischen Patientenakte. Die Erhebung erfolgt dabei für stationäre Patienten im Rahmen des Pflegeaufnahmegespräches. Vor Einführung des Fragebogens wurde das gesamte Pflegepersonal fachkompetent durch Mitarbeiter der Psychosomatik geschult. Hierbei wurde die einfache Erhebung durch bloßes Vorlesen der Frage- und Antwortmöglichkeiten sowie der Umgang mit möglichen Problemen trainiert. Die Antworten des Patienten werden mittels einer entsprechenden Maske direkt in die elektronische Akte eingegeben. Gab es anfangs nur eine Version des Fragebogens in deutscher Sprache, so kann mittlerweile bei Bedarf eine englische, türkische, arabische, polnische und russische Version ausgedruckt und dem Patienten vorgelegt werden. Darüber hinaus lassen sich evtl. Gründe wie eine Demenz oder motorische Probleme, die eine Erhebung unmöglich machen, dokumentieren (Abb. 1).

Wird durch den PHQ-4-Fragebogen ein Cut-off-Wert von mindestens 4 für Ängstlichkeit oder Depression erreicht, spricht dies für eine mindestens mittelschwere Belastung. In diesem Fall wird bei Eingabe in die elektronische Patientenakte durch das Pflegepersonal automatisch ein Feld aktiviert, das nach Unterstützungsbedarf fragt. Wenn der Patienten diesen wünscht, wird durch eine entsprechende Markierung des Feldes automatisch ohne vorherige Abstimmung mit dem Stationsarzt ein psychosomatisches Konsil generiert.

**Figure 2:**
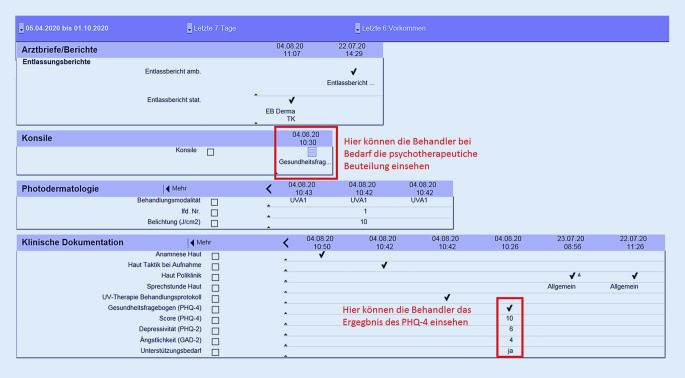

Das entsprechende psychosomatische Konsil erfolgt dann in der Regel am Folgetag und erscheint sodann ebenso wie das Ergebnis des PHQ‑4 in der digitalen Patientenakte des Patienten, sodass es jedem Behandler ersichtlich ist (Abb. 2). Zur besseren Vernetzung wurde ein eigener Raum für die psychotherapeutischen Gespräche auf der dermatologischen Station eingerichtet werden.

Ergibt sich aus dem Konsil der Bedarf einer psychosomatischen Weiterbehandlung oder ggf. sogar einer Verlegung in eine psychiatrische Einrichtung, so kann diese durch den behandelnden Stationsarzt in die Wege geleitet werden.

## Ergebnisse

Durch die Implementierung des Screenings wurden im Jahr 2019 im Bereich der stationären, dermatologischen Versorgung 83 % aller Patienten mittels PHQ-4-Fragebogen untersucht. Dies entsprach insgesamt 3060 Patienten. Bei 99 Patienten zeigte sich ein auffälliges Ergebnis, entsprechend 3,2 % aller gescreenten Patienten; 98 dieser Patienten wünschten diesbezüglich professionelle Unterstützung, sodass insgesamt 98 psychosomatische Konsile generiert wurden. Im Bereich der teilstationären Versorgung wurden 2019 alle 146 Patienten gescreent. Hierbei zeigte sich bei 49 Patienten ein auffälliges Ergebnis im PHQ‑4 (34 % der untersuchten Patienten), und 47 Patienten wünschten und erhielten auch ein psychosomatisches Konsil.

Darüber hinaus wurde bei diversen Patienten eine psychotherapeutische Behandlung über den stationären Aufenthalt hinaus eingeleitet. Bei 2 Patienten zeigte sich im Rahmen des psychosomatischen Konsils sogar eine akute Suizidalität mit bereits konkreten Ideen für einen Suizid. Die Patienten wurden sodann umgehend auf eine geschützte psychiatrische Station verlegt.

## Diskussion

Schätzungsweise 30 % aller dermatologischen Patienten leiden unter einer psychischen Komorbidität [4]. Bevor eine adäquate Therapie eingeleitet werden kann, besteht die Herausforderung, im stressigen Klinikalltag die psychischen Begleiterkrankungen zu detektieren [3]. Besondere Relevanz hat dies für Patienten mit Dermatosen wie etwa einer atopischen Dermatitis, Prurigo, Psoriasis oder auch einem Herpes zoster, die durch Stress getriggert werden und selber wiederum Stress verursachen.

In unserer Klinik ist es uns durch die Implementierung des PHQ-4-Fragebogens gelungen, Patienten mit geringem zeitlichem Aufwand bezüglich Depressivität und Ängstlichkeit zu untersuchen. Um ein valides Ergebnis über die Belastung im häuslichen Alltag zu gewährleisten, erfolgte das Screening im Rahmen der Aufnahmeuntersuchung. Mithilfe der technischen Möglichkeiten der elektronischen Akten konnte darüber hinaus eine direkte therapeutische Konsequenz in Form eines psychosomatischen Konsils ermöglicht werden.

Die Einführung des Screenings war anfänglich schwierig, da die Mitarbeiter/innen zunächst einen großen Mehraufwand sahen. Weiterhin fiel es ihnen schwer, die Fragen für den Fragebogen genau so zu stellen, wie sie in dem Bogen aufgeführt sind. Erst im Verlauf und durch gute Begleitung in Form von wiederholten Schulungen wurde der Benefit klar. Inzwischen gehört der Bogen zum Selbstverständnis der Klinik und erfreut sich aufgrund des spürbaren Nutzens großer Beliebtheit.

In unserem stationären Patientenkollektiv ergab sich bei nur 3,2 % der Patienten ein auffälliger Wert im PHQ-4-Fragebogen. Dieser verhältnismäßig geringe Prozentsatz lässt sich dabei durch die Fokussierung auf dermatoonkologische und operative Behandlungen sowie die Versorgung von notfallmäßigen, akuten Dermatosen in unserer stationären Behandlung erklären. Im Bereich der tagesklinischen Behandlung, in der vorwiegend chronische Erkrankungen mit bekannter psychischer Komorbidität wie Psoriasis, Prurigo und die atopische Dermatitis behandelt werden, zeigte sich bei 34 % der Untersuchten ein auffälliges Ergebnis im PHQ-4-Screening. Dieser Wert liegt somit knapp oberhalb des Erwartungswertes.

## Schlussfolgerung

Ein flächendeckendes Screening auf psychische Komorbiditäten in der stationären und teilstationären Versorgung von somatischen Patienten, wie es in der Dermatologie des UKEs (Universitätsklinikum Hamburg Eppendorf) erfolgt, ist nach unserem Wissensstand in Deutschland einzigartig. Aufgrund der einfachen Durchführung bei hohem Nutzen empfehlen wir die Implementierung eines Screenings mittels PHQ-4-Fragebogens in jeder dermatologischen Klinik.

Es bleibt abzuwarten, ob sich hierdurch in Zukunft neben der spürbar besseren klinischen Versorgung evtl. auch eine höhere Fallpauschale erzielen lässt. Außerdem sollte auch eine Etablierung des Screenings im ambulanten Bereich angestrebt werden.

## Fazit für die Praxis

- Ein Screening auf psychische Komorbiditäten mittels Patient Health Questionnaire‑4 (PHQ-4) ist einfach und effektiv.
- Das Wissen um eine psychische Begleiterkrankung ermöglicht in der Dermatologie eine zielgerichtete, ganzheitliche Therapie.
- Das Screening kann die dermatologische Intervention verbessern und die Patientenadhärenz fördern.
- Das Screening hat evtl. wirtschaftliche Vorteile.
